# The Treatment of Tabes

**Published:** 1898-05-28

**Authors:** 


### THE TREATMENT OF TABES.

For some years past Dr. Frenkel, of Heiden, in Switzerland, has from time to time published in various foreign periodicals accounts of a method of treating tabes devised by him. This has attracted considerable attention on the Continent, and has been favourably reported on by such well-known authorities as Leyden, Erb, Bechterew, Grasset, and Raymond; it was also the subject of a special discussion at last year's Moscow Congress. Hitherto, however, it has received but little attention in England, although at least one well-known neurologist has had good results with it in his private practice. Dr. Frenkel has recently been on a visit to England, and on May 16th he gave a demonstration at the National Hospital for the Paralysed and Epileptic, at which there were present Dr. Hughlings Jackson, Dr. Buzzard, and several other members of the staff, and a few more physicians interested in the subject. Dr. Frenkel laid great stress on the exact diagnosis of the extent and quality of the ataxy, and also on the estimation of the muscular flaccidity observed in the disease, to which he gives the name of "hypotonie musculaire." To this phenomenon he has paid particular attention; it may be noted that it corresponds to the loss of muscular tone discovered by Mott and Shenington after section of the posterior roots of spinal nerves. The main aim of Dr. Frenkel's method is the systematic education of the affected muscles by means of carefully graduated exercises. The apparatus he showed was somewhat complex, but ,as he himself explained, equally good results can be obtained in ordinary practice by much simpler means. Each individual movement is carefully and thoroughly taught, so that the treatment of each case must be on its own merits. The process of education is repeated from two to four times a day for periods of five to ten minutes. Fatigue is always to be most carefully avoided, and the "dose" of the treatment must be most accurately determined and graduated. Special attention is also given to the ataxy of the upper limbs when this is present, and this is corrected by appropriately devised apparatus. It is claimed that an ordinary case of inco-ordination is usually greatly improved in a fortnight, and often permanently cured in a month. Whether such admirable results are to be attained in England has yet to be seen, but we have no doubt that the method will receive a thorough trial, as it aims at the alleviation of one of the most distressing symptoms of a prolonged and incurable disease.

